# Eradication of Biofilm-Like Microcolony Structures of *Borrelia burgdorferi* by Daunomycin and Daptomycin but not Mitomycin C in Combination with Doxycycline and Cefuroxime

**DOI:** 10.3389/fmicb.2016.00062

**Published:** 2016-02-10

**Authors:** Jie Feng, Megan Weitner, Wanliang Shi, Shuo Zhang, Ying Zhang

**Affiliations:** Department of Molecular Microbiology and Immunology, Bloomberg School of Public Health, Johns Hopkins University, BaltimoreMD, USA

**Keywords:** *Borrelia burgdorferi*, persister, anti-persister activity, biofilm, drug combination

## Abstract

Lyme disease, caused by *Borrelia burgdorferi*, is the most common vector-borne disease in the United States and Europe. While the majority of Lyme disease patients can resolve their symptoms if treated promptly, 10–20% of patients suffer from prolonged symptoms called post-treatment Lyme disease syndrome (PTLDS). Although the cause for PTLDS is unclear, one possibility is the presence of bacterial persisters not effectively cleared by the current Lyme antibiotics. Recent studies identified several drug candidates including daptomycin, daunomycin, doxorubicin, and mitomycin C that had good activity against *B. burgdorferi* persisters. However, their relative activities against *B. burgdorferi* persisters have not been evaluated under the same conditions. In this study, we tested the anti-persister activities of these drugs against both 7-day and 15-day old stationary phase cultures of *B. burgdorferi* individually as well as in combination with Lyme antibiotics doxycycline and cefuroxime (Ceftin). Our findings demonstrate daunomycin and daptomycin were more active than mitomycin C in single drug comparison at 10 and 20 μM, as well as in drug combinations with doxycycline and cefuroxime. In addition, daunomycin was more active than doxorubicin which correlated with their ability to stain and accumulate in *B. burgdorferi.* The two drug combination of doxycycline and cefuroxime was unable to eradicate biofilm-like microcolonies of *B. burgdorferi* persisters. However, the addition of either daunomycin or daptomycin to the doxycycline + cefuroxime combination completely eradicated the biofilm-like structures and produced no visible bacterial regrowth after 7 and 21 days, while the addition of doxorubicin was unable to prevent regrowth at either 7 or 21 day subculture. Mitomycin C in combination with doxycycline and cefuroxime caused no regrowth at 7 days but visible spirochetal regrowth occurred after 21 day subculture. Furthermore, we found that cefuroxime (Ceftin), the third commonly used and most active antibiotic to treat Lyme disease, could replace cefoperazone (a drug no longer available in the US) in the daptomycin + doxycycline combination with complete eradication of the biofilm-like structures as shown by lack of any regrowth in subcultures. Our findings may have implications for improved treatment of Lyme disease.

## Introduction

*Borrelia burgdorferi* is the causative agent of Lyme disease, which is the most common vector-borne disease in the United States with an estimated 300,000 cases in 2013(CDC, 2015a). The infection is transmitted to humans by tick vectors that feed upon rodents, reptiles, birds and deer (Radolf et al., 2012). In the early stage of Lyme disease, approximately 50% of patients have localized erythema migrans, a target-shaped rash that expands as the bacteria disseminate from the cutaneous infection site (CDC, 2015a). Late stage Lyme disease is a multi-system disorder with symptoms including arthritis, carditis, and neurologic impairment (CDC, 2015a). The majority of Lyme disease patients can resolve their symptoms if treated promptly with doxycycline, amoxicillin, or cefuroxime (Wormser et al., 2006). However, at least 10–20% of Lyme disease patients experience prolonged symptoms such as neurologic impairment, muscular pain, and fatigue 6 months after antibiotic treatment, a collection of symptoms called Post-Treatment Lyme Disease Syndrome (PTLDS; CDC, 2015b).

The cause of PTLDS is unknown, though there are several theories including co-infections (Swanson et al., 2006), autoimmune response (Steere et al., 2001), immune response to continued presence of antigenic debris (Bockenstedt et al., 2012), as well as *B. burgdorferi* persisters that are not killed by the current antibiotics (Hodzic et al., 2008, 2014; Embers et al., 2012). Using a combination of diagnostic techniques including xenodiagnosis and PCR, studies have found evidence of *B. burgdorferi* persistence in dogs (Straubinger et al., 1997), mice (Hodzic et al., 2008, 2014), monkeys (Embers et al., 2012), and humans (Marques et al., 2014) after antibiotic treatment, though no viable bacteria could be cultured.

*Borrelia burgdorferi* develops persisters stochastically in stationary phase which are tolerant to the antibiotics used to treat Lyme disease (Feng et al., 2014a, 2015a; Caskey and Embers, 2015; Sharma et al., 2015). These persister bacteria have been found to have an altered RNA profile, making them phenotypically drug tolerant (Feng et al., 2015c). In log phase cultures (3–5 days old), *B. burgdorferi* is primarily in motile spirochetal form which is highly susceptible to current Lyme antibiotics doxycycline and amoxicillin, however, in stationary phase cultures (7–15 days old), increased numbers of atypical forms such as round bodies and aggregated biofilm-like microcolonies develop (Feng et al., 2014a, 2015a). These atypical forms have been shown to have increased tolerance to doxycycline and amoxicillin when compared to the growing spirochetal forms (Feng et al., 2014a, 2015a; Caskey and Embers, 2015; Sharma et al., 2015). Therefore, stationary phase cultures (7–15 days old) which are enriched in persisters were used as a model for high-throughput drug screens against persister populations (Feng et al., 2014a, 2015a,b,d).

Drugs with high activity against the *B. burgdorferi* stationary phase persisters were identified through screens of FDA approved drug library and NCI compound libraries (Feng et al., 2014a, 2015b,d). Among them, daptomycin, a lipopeptide antibiotic targeting bacterial cell membranes, was found from the FDA drug library to have the highest anti-persister activity against *B. burgdorferi* (Feng et al., 2014a). In addition, anticancer anthracycline antibiotics, such as daunomycin and doxorubicin, and also mitomycin C were found from the NCI compound library screen as having excellent or good activity against *B. burgdorferi* persisters (Feng et al., 2015b). Daunomycin, doxorubicin and mitomycin C were all isolated from *Streptomyces* and are used in the treatment of a wide range of cancers. Daunomycin and doxorubicin belong to anthracycline anti-cancer antibiotic and kill the bacteria by inhibiting DNA and RNA synthesis, causing DNA damage and producing reactive oxygen species. Mitomycin C blocks DNA replication and causes cell death by DNA crosslinking. Although the anti-persister drugs such as daptomycin are more active than the current Lyme antibiotics such as doxycycline or amoxicillin against *B. burgdorferi* persisters (Feng et al., 2014a), they alone could not completely eradicate the more resistant biofilm-like microcolonies and a drug combination approach is required to do so (Feng et al., 2015a). The more effective drug combination approach to eradicate biofilm-like microcolonies is consistent with the drug combination principle for treatment of persistent infections like tuberculosis (Zhang et al., 2012; Zhang, 2014). In a recent study using a relatively young 5 days old culture, mitomycin C was found to have higher activity than daptomycin against *B. burgdorferi* persisters (Sharma et al., 2015). However, their relative activity against *B. burgdorferi* persisters has not been compared or evaluated in the same study under the same conditions. In this study, four of the identified drugs with the highest activity against stationary phase *B. burgdorferi* persisters were tested to determine their anti-persister activity at more clinically achievable levels. In addition, we assessed these persister active drugs in combination with the commonly prescribed Lyme antibiotics doxycycline and cefuroxime, which have high activity against growing log phase cultures, with the aim to increase the activity of these drugs for more effective treatment of Lyme disease.

## Materials and Methods

### Strain, Media, and Culture Techniques

*Borrelia burgdorferi* strain B31 (ATCC 35210) was obtained from American Type Tissue Collections (Manassas, VA, USA). *B. burgdorferi* was grown in BSK-H medium (HiMedia Laboratories, Mumbai, India) and supplemented with 6% rabbit serum (Sigma Aldrich, St. Louis, MO, USA). The medium was filter-sterilized via passage through a 0.22 μM filter. The inoculated medium was incubated in sterile 50 mL conical tubes (BD Biosciences, San Jose, CA, USA) in a 33°C incubator without shaking. The culture was maintained in these conditions for 7 or 15 days until the culture reached stationary phase, when it was transferred to a 96 well plate for evaluation with the drugs or their combinations.

### Drugs

The following drugs were obtained from Sigma–Aldrich, St. Louis, MO, USA and dissolved in the solvents suggested by the Clinical and Laboratory Standards Institute to make a stock solution: doxycycline (Dox), cefuroxime (CefU), cefoperazone (CefP), daptomycin (Dap), mitomycin C (MitC), doxorubicin (DoxR), daunomycin (Dau), (Clinical and Laboratory Standards Institute, 2007). The drug stock solutions were filter-sterilized using a 0.22 μM filter and stored at –20°C.

### Microscopy

The *B. burgdorferi* cultures were examined using a Zeiss AxioImager M2 microscope with differential interference contrast and epifluorescent illumination. Pictures were taken using a SPOT slider camera. A SYBR Green I/PI assay was performed as previously described to assess cell viability using the ratio of green:red fluorescence to determine the live:dead cell ratio, respectively, as measured by a plate reader (Feng et al., 2014b). This residual cell viability reading was confirmed by analyzing three representative images of the bacterial culture using epifluorescence microscopy. Image Pro-Plus software was used to quantitatively determine the fluorescence intensity.

### Evaluation of Drugs Against Biofilm-Like Structures in *B. burgdorferi* Stationary Phase Cultures

For single drug evaluation, an aliquot of the drug stock solution was added to each 96 well plate containing 100 μL of 7-day old stationary phase *B. burgdorferi* culture to obtain the desired drug concentration. The plate was then sealed and was incubated at 33°C without shaking for 7 days. After incubation, the viability of the residual viable cells was assessed using the SYBR Green I/PI viability assay and confirmed using epifluorescence microscopy (Feng et al., 2014b). Each sample was analyzed in triplicate and the mean residual viable cells remaining were calculated.

For assessing the activity of anthracycline compounds and daptomycin and mitomycin C in combination with current Lyme antibiotics against biofilm-like structures, a 15-day old *B. burgdorferi* stationary phase culture was used. Aliquots of the drugs were added to 96 well plate containing 100 μL of the 15-day old stationary phase *B. burgdorferi* culture which was enriched in aggregated biofilm-like structures to create a final drug concentration of 10 μg/mL for each drug. This drug concentration was chosen as most drugs evaluated in this study fell within or close to their *C*_max_ values (maximum serum concentration; **Table 1**). The plate was then sealed and was incubated at 33°C without shaking for 7 days, when the residual viable cells remaining were measured using the SYBR Green I/PI viability assay and confirmed using epifluorescence microscopy as described (Feng et al., 2014b).

**Table 1:** Relative activity of daunomycin, daptomycin, doxorubicin, and mitomycin C on a 7-day old *B. burgdorferi* stationary phase culture^a^.

Drugs	0 μM	5 μM	10 μM	20 μM	*C*_max_
Daunomycin	89%	60%	50%	20%	30.2 μg/mL (57 μM)
Daptomycin	89%	80%	60%	38%	57.8–93.9 μg/mL (36–58 μM)
Doxorubicin	89%	88%	66%	50%	4.12–8.34 μg/mL (7.6–15.3 μM)
Mitomycin C	89%	88%	85%	77%	2.4 μg/mL (7.2 μM)


*^a^A 7 days old stationary phase culture of *B. burgdorferi* was treated with the indicated drugs at different concentrations (0, 5, 10, and 20 μM) for 7 days. Residual viability of *B. burgdorferi* after the antibiotic treatment was determined using the SYBR Green I/PI viability assay followed by epifluorescence microscopy and calculation of the percentage of green (viable) cells over red (dead) cells as described (Feng et al., 2014a). Viabilities are the average of three replicates.*

### Subculture Study to Assess the Effect of Drug Combination on the Biofilm-Like Structures in *B. burgdorferi* Stationary Phase Cultures

A 15-day old *B. burgdorferi* culture (500 μL of 1 × 10^7^ spirochetes/mL) was exposed to the indicated drug combinations in Eppendorf tubes, and incubated at 33°C for 7 days without shaking. After incubation, the bacteria were spun down and washed with 1 mL fresh BSK-H medium. The cultures were resuspended in 500 μL BSK-H medium, and a 50 μL aliquot was used to inoculate a new tube of 1 mL fresh BSK-H medium for subculture. The cultures were allowed to grow for either 7 or 21 days, when they were evaluated for regrowth with viable cells using the previously described SYBR Green I/PI assay and epifluorescence microscopy (Feng et al., 2015a).

## Results and Discussion

### Comparison of the Relative Anti-Persister Activity of Daunomycin, Doxorubicin, Daptomycin, and Mitomycin C in Single Drug Exposure Against Stationary Phase *B. burgdorferi* Culture

**FIGURE 1:**
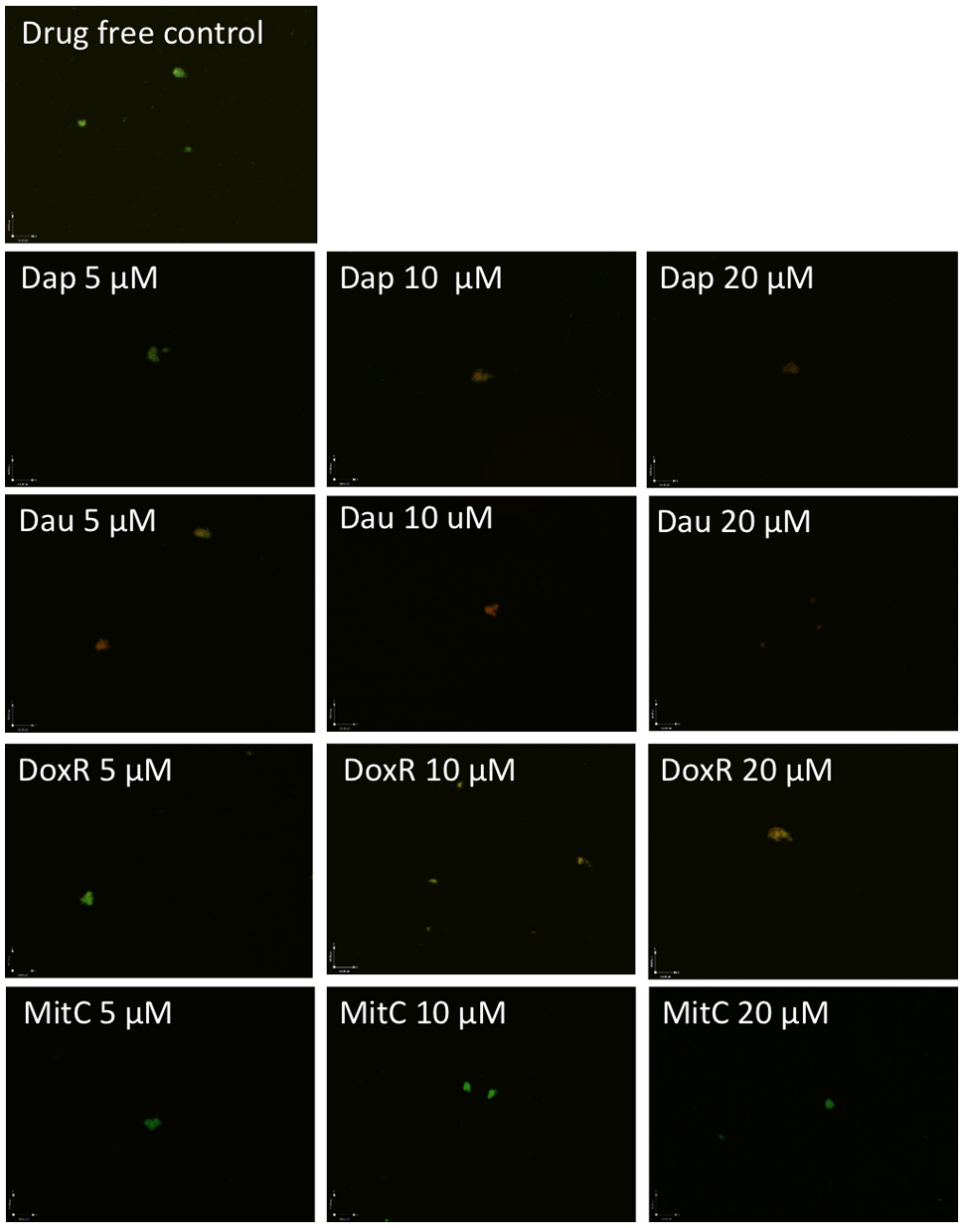
**Comparison of anti-persister activity of daunomycin, doxorubicin, daptomycin, and mitomycin C.** A 7-day old *B. burgdorferi* stationary phase culture containing aggregated microcolonies was incubated for 7 days with daptomycin (Dap), daunomycin (Dau), doxorubicin (DoxR), or mitomycin C (MitC) at the same drug concentrations of 5, 10, or 20 μM, respectively, followed by viability assessment using the SYBR Green I/PI assay. Representative images were taken using epifluorescence microscopy at 400× magnification. Green cells indicate live cells while red cells indicate dead cells.

Although daptomycin (Feng et al., 2014a), daunomycin (Feng et al., 2015b), doxorubicin (Feng et al., 2015b), and mitomycin C (Feng et al., 2015b; Sharma et al., 2015) were identified to have high activity against *B. burgdorferi* persisters, their relative activities have not been compared under the same conditions. To do so, we compared them for their activity against the same 7-day old *B. burgdorferi* stationary phase culture at the same concentrations (5, 10, and 20 μM), using SYBR Green I/PI viability stain followed by epifluorescence microscopy. The anthracycline drug daunomycin was shown to have the highest activity against the stationary phase cultures even at the lowest concentration (5 μM) with a dose-dependent increase in killing activity resulting in a near total clearance of bacteria at the highest concentration (20 μM) as shown by mostly red (dead) cells and dispersed, smaller aggregated microcolony size, revealed by the SYBR Green I/PI viability assay (**Figure 1**, **Table 1**). Daptomycin was the second most active drug against the *B. burgdorferi* stationary phase culture, followed by doxorubicin (**Figure 1**, **Table 1**). Mitomycin C was the least active drug among the four persister-active drugs, and even at 20 μM had poor activity against the aggregated biofilm-like microcolony form of *B. burgdorferi* persisters, as shown by mostly green (live) microcolonies remaining after the drug treatment for 7 days (**Figure 1**).

**FIGURE 2:**
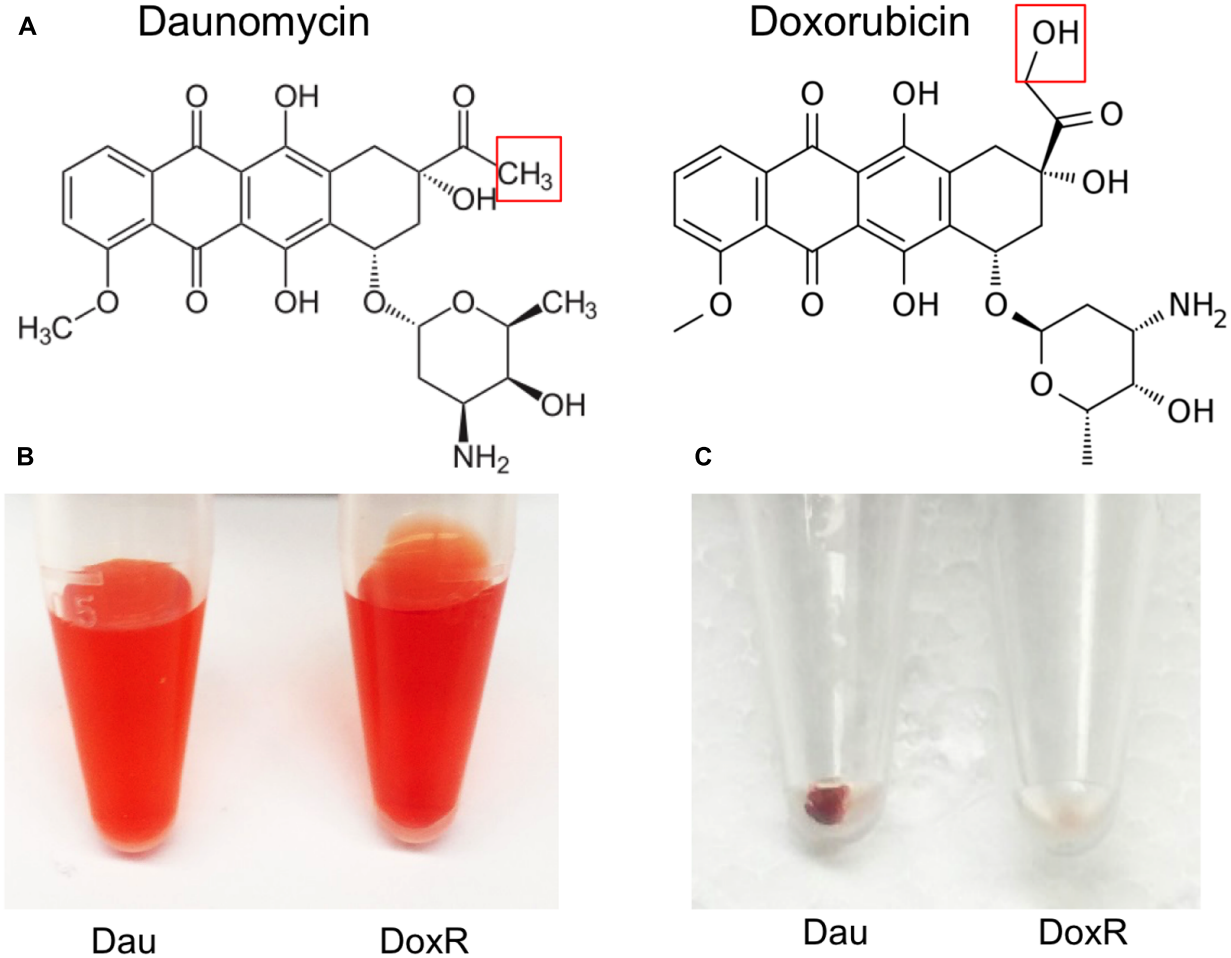
**Differences in structures of daunomycin and doxorubicin and their ability to accumulate in *B. burgdorferi*.** **(A)** Chemical structures of daunomycin and doxorubicin. Red box shows the difference between the structures of daunomycin (methyl group) and doxorubicin (hydroxyl group). **(B)** Daunomycin and doxorubicin show the same orange–red color at 10 mM solution. **(C)** Cell pellets of 7-day old *B. burgdorferi* treated with 10 μM daunomycin (left-side tube) 10 μM doxorubicin (right-side tube) for 7 days, where daunomycin stained *B. burgdorferi* red while doxorubicin hardly stained the organism.

Doxorubicin was less active than daunomycin as shown by higher percentage of viable cells remaining after drug exposure (**Table 1**) despite their both belonging to the same anthracycline class. These results could be explained by structural differences between those compounds (**Figure 2A**). Doxorubicin possesses a hydroxyl group as opposed to a methyl group in the corresponding position of daunomycin, with the remainder of the anthracycline structure being identical. Interestingly, although doxorubicin and daunomycin both have orange-red color in solution (**Figure 2B**), we found daunomycin visibly stained the *B. burgdorferi* cells red as seen in the red cell pellet while doxorubicin only stained the cells rather faintly (**Figure 2C**). This finding suggests that daunomycin may cross the *B. burgdorferi* cell membrane more efficiently to accumulate in the cell while doxorubicin may have poor ability to enter or accumulate in *B. burgdorferi* cells.

### Comparison of the Relative Anti-Persister Activity of Daunomycin, Daptomycin, Doxorubicin, and Mitomycin C in Drug Combinations Using SYBR Green I/PI Viability Assay

**FIGURE 3:**
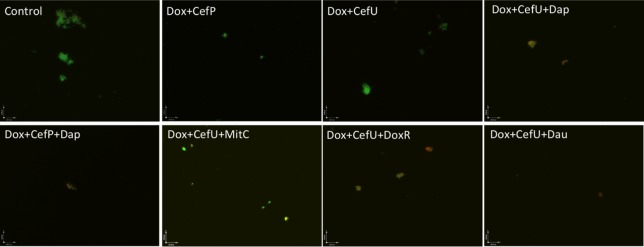
**Comparison of the activity of daunomycin, daptomycin, and mitomycin C in combination with currently used Lyme antibiotics.** A 15-day old *B. burgdorferi* stationary phase culture was incubated with the indicated drug combinations at a final concentration of 10 μg/mL for each antibiotic for 7 days followed by SYBR Green I/PI stain and epifluorescence microscopy. Abbreviations: Dox, doxycycline; CefU, cefuroxime; CefP, cefoperazone; Dap, daptomycin; MitC, mitomycin C; DoxR, doxorubicin; Dau, daunomycin.

Both two-drug combinations doxycycline + cefuroxime and doxycycline + cefoperazone showed poor activity against the 15-day old stationary phase culture, with 67% residual viable (green) cells remaining in comparison to 79% viable cells in the drug-free control (**Figure 3**, **Table 2**). Consistent with the single drug exposure experiment (**Figure 1**), daunomycin, doxorubicin and daptomycin when added to the drug combination doxycycline + cefuroxime had a high anti-persister activity as seen by 12, 18, and 30% viable cells remaining (**Table 2**) as well as mostly red (dead) cells after treatment (**Figure 3**). In contrast, when mitomycin C was added to the drug combination doxycycline + cefuroxime, the anti-persister activity of these compounds was moderately increased as shown by 45% residual viable cells remaining (**Table 2**), but more green (live) cells were seen with the mitomycin C drug combination than with the daunomycin or daptomycin drug combination (**Figure 3**).

**Table 2:** Viability of stationary phase *B. burgdorferi* after antibiotic treatment^a^ assessed by direct SYBR Green I/PI viability assay and subculture.

Drugs	Residual viable cells^b^	Number of spirochetes after 7 days subculture	Number of spirochetes after 21 days subculture
Drug-free control	79%	6 × 10^6^	2 × 10^7^
Dox + Cefu	67%	1 × 10^6^	1 × 10^7^
Dox + Cefp	67%	9 × 10^5^	1 × 10^7^
Dox + Cefu + MitC	45%	0^c^	1 × 10^6^
Dox + Cefu + DoxR	18%	4 × 10^5^	8 × 10^5^
Dox + Cefu + Dap	30%	0^c^	0^c^
Dox + Cefp + Dap	29%	0^c^	0^c^
Dox + Cefu + Dau	12%	0^c^	0^c^


*^a^A 15-day old stationary phase *B. burgdorferi* culture (1 × 10^7^ spirochetes) was treated with 10 μg/mL of each antibiotic for 7 days. After treatment, 50 μL of the washed bacteria were inoculated into 1 mL fresh BSK-H medium and cultured for 7 or 21 days, when any possible regrowth was assessed by SYBR Green I/PI staining followed by epifluorescence microscopy. ^b^Residual viable cells after 7 days antibiotic treatment were calculated using epifluorescence microscopy as previously described (Feng et al., 2014a). ^c^Values are below the limit of detection of <1 × 10^5^ by microscopy.*

### Subculture Study to Assess the Relative Anti-Persister Activity of Daunomycin, Daptomycin, and Mitomycin C in Drug Combinations

**FIGURE 4:**
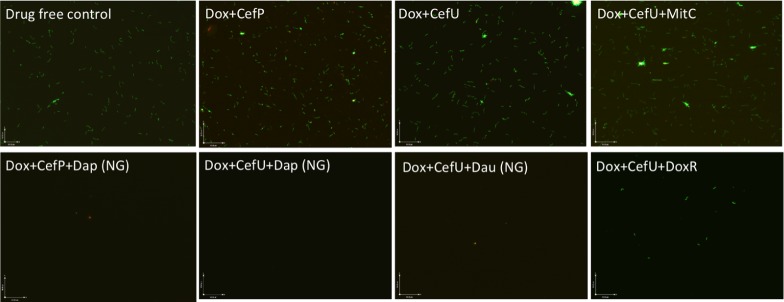
**Subculture (21 days) of 15-day old B. burgdorferi stationary phase culture treated with different drug combinations.** The 15-day old *B. burgdorferi* culture was incubated with the indicated drug combinations at a final concentration of 10 g/mL for each antibiotic for 7 days followed by washing and resuspension of cells in fresh BSK medium and subcultured for 21 days. The viability of the subculture was examined by SYBR Green I/PI viability assay and epifluorescence microscopy (400 magnification). NG, no growth.

To validate the activity of these drug combinations, samples of the above drug-treated cultures were subjected to subculture after removal of the drugs by washing followed by incubation in fresh BSK medium for 7 or 21 days. A lack of visible regrowth when measured by microscopy suggests that few to no viable cells remain after drug treatment, while visible regrowth of the culture indicates the presence of viable cells after drug treatment. The addition of daunomycin or daptomycin to the doxycycline + cefuroxime drug combination showed no visible regrowth after 7 and 21 days, suggesting no viable *B. burgdorferi* organisms were left after the treatment (**Figure 4**). Despite the high anti-persister activity of doxorubicin + doxycycline + cefuroxime in the microscopic analysis (**Figure 3**), with only 18% residual viable cells after treatment (**Table 2**), this triple drug combination was unable to prevent bacterial regrowth at either 7 or 21 days subculture, indicating it is not as active as daunomycin or daptomycin (**Table 2**, **Figure 4**). However, doxorubicin + doxycycline + cefuroxime was more active than mitomycin C + doxycycline + cefuroxime as shown by less regrowth than the latter combination (**Figure 4**). This is consistent with the single drug data where doxorubicin was more active than mitomycin C (**Figure 1**).

The discrepancy in the activity of doxorubicin in epifluorescence microscopy based viability analysis and subculture study was noted in our previous studies (Feng et al., 2015a,b). This is due to the red orange color of the anthracycline drug doxorubicin, which stains the cells red and could give false impression of a high killing activity. However, subculture studies were able to show the inability of doxorubicin + doxycycline + cefuroxime to eradicate the microcolony form of *B. burgdorferi* persisters as shown by regrowth after subculture. Thus, the subculture study is crucial in validating the results of other forms of viability assays such as SYBR Green I/PI assay in persister drug evaluations.

When mitomycin C was added to doxycycline + cefuroxime combination treated *B. burgdorferi* stationary phase culture, there was no regrowth at 7 days, but visible spirochetal regrowth occurred after 21 days subculture (**Figure 4**). This finding suggests that the addition of mitomycin C to the commonly used Lyme antibiotics doxycycline + cefuroxime is not as active as the addition of daunomycin or daptomycin, but is more active than doxorubicin. Furthermore, this result indicates that 7 days subculture is not sufficient to reveal the small number of residual bacteria remaining after drug treatment and that a prolonged incubation to 21 days is needed to demonstrate the small numbers of viable bacteria for more reliable evaluation of drug combinations against *B. burgdorferi* persisters.

In a recent study, mitomycin C was shown to be more active than daptomycin and to eradicate all *B. burgdorferi* persisters (Sharma et al., 2015). This is in contrast to the results of this study which found daptomycin to have higher anti-persister activity than mitomycin C in both single drug exposure (**Table 1**, **Figure 1**) and drug combination studies (**Figures 3** and **4**). Several possibilities exist to explain the discrepancy. First, we used older 7 and 15 days stationary phase culture*s* containing an increased number of persisters and biofilm-like microcolonies previously shown to have increased tolerance to antibiotics (Feng et al., 2015a), while the other study used a younger culture of 5 days (Sharma et al., 2015), which would have more growing cells and fewer persister cells. The difference in persister numbers in these cultures would result in the bacteria in the younger culture of 5 days being more easily killed by mitomycin C but not by daptomycin. Indeed, daptomycin is known to have relatively high MIC (12.5–25 mg/mL) for growing spirochetes despite its high activity against *B. burgdorferi* persisters (Feng et al., 2014a), and this may also explain why daptomycin had limited activity in that study as a younger culture was used (Sharma et al., 2015). The use of a younger culture in the other study that contained mainly growing spirochetes is also consistent with their finding that the 5-day old culture was readily killed by even amoxicillin and ceftriaxone (Sharma et al., 2015), which are known to kill mainly growing bacteria. Second, we used different viability assays. In this study, we used SYBR Green I/PI viability stain along with microscopy and subculture in liquid medium to assess the viability of residual bacteria after drug treatment. In contrast, the other study used colony forming unit (CFU) assay on solid agar to determine the viable bacteria after drug exposure (Sharma et al., 2015). Based on studies with other bacteria like *M. tuberculosis* (Dhillon et al., 2004), the CFU assay favors the detection of more viable organisms and is less sensitive than culture in liquid medium which can detect small numbers of viable cells which may not grow on solid medium after drug exposure. Third, we used BSK-H medium, which is richer than the BSK-II medium used by the other study (Sharma et al., 2015), which might have also contributed to the higher activity of mitomycin C than daptomycin in that study (Sharma et al., 2015).

### Cefuroxime (Ceftin) Could Replace Cefoperazone in the Daptomycin + Doxycycline Combination to Completely Eradicate Biofilm-Like Structures

In our previous drug combination study, we found that daptomycin + doxycycline + cefoperazone was able to completely eradicate the most resistant aggregated biofilm-like microcolonies (Feng et al., 2015a). However, since cefoperazone is not available in the US, we replaced it with the current Lyme antibiotic cefuroxime (Ceftin) in the daptomycin + doxycycline combination and found they had equivalent activity as shown by the same 30% residual viable cells after antibiotic treatment using SYBR Green I/PI viability stain and microscopy (**Figure 3**, **Table 2**). In subculture studies, we found replacement of cefoperazone with cefuroxime (Ceftin) in the daptomycin + doxycycline combination similarly resulted in complete eradication of the biofilm-like structures as shown by lack of any regrowth in 7 and 21 days subcultures (**Figure 4**).

Cefuroxime and cefoperazone, which are second and third generation cephalosporins, respectively, function as a highly penetrative beta-lactam antibiotic by disrupting the bacterial cell wall biosynthesis (Barriere and Flaherty, 1984). Despite being reported as the best beta-lactam antibiotic in the 7-day stationary phase persister model (Feng et al., 2014a), cefoperazone did not give any advantage over the use of the commonly prescribed Lyme antibiotic cefuroxime (Ceftin) in the context of drug combination with daptomycin + doxycycline (**Figures 3** and **4**). This data suggests that replacing cefoperazone with the commonly used cefuroxime (Ceftin) will maintain comparable efficacy against the biofilm-like microcolony form of *B. burgdorferi* persisters in the drug combination with daptomycin + doxycycline.

In this study, we were able to confirm our previous observations of the high anti-persister activity of daptomycin (Feng et al., 2014a, 2015a) and daunomycin (Feng et al., 2015b) alone and in drug combination with doxycycline + cefuroxime.It is worth noting that the high anti-persister activity of daptomycin and the anthracycline antibiotic daunomycin is due to the unique mechanisms of action through disruption of cell membrane and damage of DNA, respectively (Feng et al., 2014a, 2015b). These observations suggest that bacterial membranes and DNA integrity are important targets for bacterial persister drugs. The complete eradication of biofilm-like structures of *B. burgdorferi* by daunomycin or daptomycin in drug combination with doxycycline + cefuroxime, again supports the Yin–Yang treatment principle of combining drugs that target growing bacteria (Yang; with doxycycline + cefuroxime) and drugs like daunomycin or daptomycin that target non-growing persisters (Yin) for more effective treatment of persistent infections (Zhang, 2014). This strategy may be generally useful for treatment of persistent infections including biofilm infections, which cannot be eradicated by a single drug alone. Future studies are needed to validate this principle.

Although our findings that daunomycin or daptomycin plus doxycycline + cefuroxime could completely eradicate the biofilm-like structures are encouraging, they are *in vitro* studies and have limitations and cannot be equated to the clinical situation. Moreover, daunomycin and daptomycin are intravenous drugs and not convenient to administer. Future studies to develop oral regimens as effective as the above combinations are needed for more convenient administration. In addition, the toxicity associated with the anticancer drug daunomycin calls for caution with its use in clinical settings. Further *in vivo* animal studies are needed to validate the highly active drug combinations identified in this study before they can be used for patient treatment in the clinic.

## Conclusion

In summary, we found that daunomycin and daptomycin were more active against *B. burgdorferi* biofilm-like structures than mitomycin C and doxorubicin in single drug comparisons as well as in drug combinations. Daunomycin or daptomycin when added to doxycycline + cefuroxime completely eradicated the biofilm-like structures, while the two drug combination doxycycline + cefuroxime alone or mitomycin C and doxorubicin when added to the above combination failed to do so. Additionally, we showed that cefuroxime (Ceftin) could replace cefoperazone in the daptomycin + doxycycline combination and caused complete eradication of the biofilm-like structures. Future studies are needed to evaluate these promising drug combinations *in vivo* in animal models, and if promising, in patients. Our findings may have implications for improved treatment of Lyme disease.

## Author Contributions

YZ conceived the experiments; JF, MW, WS, SZ, performed the experiments; JF, MW, and YZ analyzed the data; and MW, JF, YZ wrote the paper.

## Conflict of Interest Statement

The authors declare that the research was conducted in the absence of any commercial or financial relationships that could be construed as a potential conflict of interest.
